# Society Meetings

**Published:** 1894-10

**Authors:** 


### Society Meetings.

The American Medical Publishers' Association held its first annual meeting at Hot Springs, Va., on August 13th and 14th. The president, Dr. Landon B. Edwards, read a paper on Advertising and Advertising Agencies, which was well received and ordered printed. The reports of the secretary and treasurer were examined, and the finances of the association found to be in good condition. Thirteen applications for membership were presented and acted upon favorably. The secretary was instructed to investigate the laws of different states governing charters of incorporated bodies, and report at the next meeting. It was decided that all annual meetings hereafter should be held just prior to the sessions of the American Medical Association, the next meeting being set for Monday, June 5, 1895, at 9 A. M., in the Eutaw House, Baltimore, Md.

---

The Southern Surgical and Gynecological Association will hold its seventh annual meeting in Charleston, S. C., Tuesday, Wednesday and Thursday, November 13, 14 and 15, 1894, under the presidency of Dr. Cornelius Kollock, of Cheraw, S. C. The secretary, Dr. W. E. B. Davis, of Birmingham, Ala., in a circular recently issued, invites attention to the importance of sending in the titles of papers at an early day. The meeting promises to be one of great interest.

---

The Tri-State Medical Society of Alabama, Georgia and Tennessee will hold its sixth annual meeting in Atlanta, Ga., Tuesday,

Wednesday and Thursday, October 9, 10 and 11, 1894. The sessions will be held in the ball-room of the Kimball House, Atlanta, which has admirable acoustic properties, and is completely shut off from all noise from the street. Dr. Frank Trester Smith, of Chattanooga, is secretary, and Dr. J. B. S. Holmes, of Atlanta, president.

---

THE Mississippi Valley Medical Association will hold its twentieth annual meeting at Hot Springs, Ark., Tuesday, Wednesday, Thursday and Friday, November, 20, 21, 22 and 23, 1894. The Hotel Eastman will be thrown open to the association for its sessions. The beautiful Indian summer season chosen for the meeting will invite a large attendance.

---

THE Buffalo Academy of Medicine, after its summer adjournment, resumed its regular meetings on Tuesday, September 11, 1894, at which time Dr. John H. Pryor read a paper on Tubercular Pneumonia, and Dr. A. L. Benedict read one with reference to the administration of opiates. Dr. De Lancey Rochester led the discussion.

---

THE Medical Society of Virginia will hold its next annual meeting in Richmond, Tuesday, Wednesday and Thursday, October 23, 24 and 25, 1894. The subject for general discussion, open to any physician registered in attendance, is appendicitis.

---

THE New York State Association of Railway Surgeons will hold its annual meeting Thursday, November 15, 1894, at the New York Academy of Medicine. All railway surgeons of the United States are invited to attend.

